# Removal of Duckbill‐type laser‐cut anti‐reflux metal stents: Clinical evaluation and in vitro study

**DOI:** 10.1002/deo2.217

**Published:** 2023-02-23

**Authors:** Yuto Yamada, Takashi Sasaki, Tsuyoshi Takeda, Takeshi Okamoto, Takafumi Mie, Chinatsu Yonekura, Takaaki Furukawa, Akiyoshi Kasuga, Masato Matsuyama, Masato Ozaka, Takahisa Matsuda, Yoshinori Igarashi, Naoki Sasahira

**Affiliations:** ^1^ Department of Hepato‐Biliary‐Pancreatic Medicine, Cancer Institute Hospital Japanese Foundation for Cancer Research Tokyo Japan; ^2^ Department of Internal Medicine Division of Gastroenterology and Hepatology Toho University, Omori Medical Center Tokyo Japan

**Keywords:** anti‐reflux metal stent, biliary, laser‐cut, self‐expandable metal stent, stent removal

## Abstract

**Objectives:**

Duckbill‐type metal stent (DMS) was the first laser‐cut biliary metal stent with an anti‐reflux valve. Removal of DMS is believed to be difficult and relevant reports are scarce. This study aims to investigate the feasibility of DMS removal.

**Methods:**

We retrospectively analyzed patients who underwent DMS removal between June 2019 and March 2022 to evaluate success rates and factors affecting outcomes. In addition, six different methods of DMS removal were reproduced in vitro, varying removal devices, angle of applied force, and grasped location. Extraction resistance, the distance of forceps stroke, and stent length after removal were compared.

**Results:**

Forty patients were enrolled, and DMS removal was successful in 31 cases (78%). No adverse events were observed. Tumor ingrowth was evident in 78% (7/9) of failed cases. Patients receiving biliary metal stents for the first time (naïve cases), long indwelling time, longer stent, and stent tearing during removal were associated with unsuccessful stent removal. In the in vitro study, a larger force was required to remove the stent at an extraction angle of 120° than at 0°. Among cases in which force was applied at 120°, the load tended to be lower when rat‐tooth forceps were applied horizontally across the stent.

**Conclusions:**

Stent removal was possible in a majority of cases. Deployment of additional stents inside DMS may be preferable to forceful removal in the presence of factors associated with difficult stent removals, such as tumor ingrowth, naïve cases, longer stents, long indwelling time, and stent tearing during removal.

## INTRODUCTION

Distal malignant biliary obstruction is a frequent complication of pancreatic cancer, biliary tract cancer, and lymph node metastases. Self‐expandable metal stents (SEMSs) have been reported to achieve longer stent patency and to be more cost‐effective than plastic stents in cases with distal malignant biliary obstruction.^1^ Covered metal stents (CMSs) are widely used because they prevent tumor ingrowth and are easier to remove than uncovered metal stents. In contrast, CMSs are more prone to stent migration and biofilm formation on the cover, resulting in stent obstruction.^2^, ^3^ SEMSs are classified into two types according to their structure: laser‐cut and braided SEMSs. Laser‐cut SEMSs have a zigzagged design with no crossing struts in the mesh, making them difficult to remove endoscopically because they tear easily when longitudinal traction is applied.^4^ However, the design reduces stent migration and facilitates accurate placement due to minimal stent shortening and low axial force.^5^, ^6^, ^7^, ^8^

**FIGURE 1:**
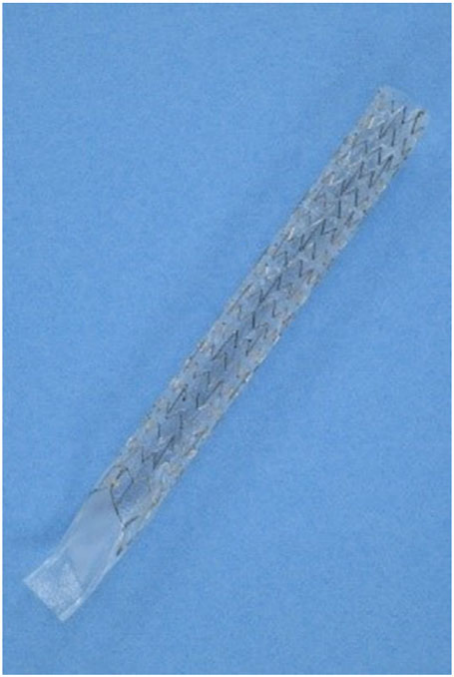
Duckbill‐type metal stents are fully‐covered, laser‐cut type self‐expandable metal stents with a 12.5 mm duckbill‐shaped anti‐reflux valve attached to the distal end (Duckbill Biliary Stent; SB‐Kawasumi Laboratories, Inc, Tokyo, Japan). The stent is made of nitinol wire and wrapped in an expanded polytetrafluoroethylene cover. Cells are arranged in a zigzagged pattern in the short‐axis direction, with three long‐axis struts connecting them together. Stents with a 10 mm diameter and lengths of 6 and 8 cm were used.

Duckbill‐type metal stents (DMSs) are fully‐covered laser‐cut SEMSs with a duckbill‐shaped anti‐reflux valve (ARV) attached to the distal end (Figure 1), shown to be effective in several reports including ours.^7^, ^8^, ^9^, ^10^, ^11^, ^12^ Their migration rates have also been reported to be lower than that of braided anti‐reflux metal stents (7%–10% vs. 16.2％).^7^, ^8^, ^13^

Given recent advances in chemotherapy, patients with unresectable cancer are surviving longer and increasingly require re‐intervention following recurrent biliary obstruction (RBO).^14^, ^15^ Removal of occluded SEMSs and replacement with new SEMSs is the ideal approach, especially in cases with favorable prognoses.^16^ However, reports on DMS removal are scarce. We conducted a retrospective study to investigate the feasibility, safety, and indications for DMS removal in the clinical setting. We also conducted an in vitro study to investigate the optimal method of DMS removal.

## METHODS

### Clinical evaluation

#### Patients

Consecutive patients with unresectable distal MBO who underwent DMS placement and required subsequent re‐intervention at our institution between June 2019 and March 2022 were enrolled in this retrospective study. Indications for stent removal included i) RBO, ii) complications of stent placement such as obstructive pancreatitis, and iii) change of biliary drainage route due to duodenal stenosis (in cases where the scope could not reach the papilla or where duodeno‐biliary reflux was a concern due to duodenal stenosis, we performed endoscopic ultrasound‐guided biliary drainage even in non‐RBO cases). Exclusion criteria included i) re‐intervention resulting from complete outward stent migration, ii) duodenal stenosis precluding access to, and removal of, indwelling DMSs, and iii) patients in whom DMS removal was not attempted due to poor general condition. Stent removal was not attempted and a plastic stent was placed in DMS in some patients in poor general condition or with prolonged clotting times.

This study was conducted in accordance with the Declaration of Helsinki and approved by the ethics committee of our institution (approval number: 2020‐GA‐1331). All patients provided written informed consent for endoscopic procedures. Consent for enrollment in this study was waived by the ethics committee due to its retrospective nature. Patients were permitted to opt out of the study without any impact on their care.

### Endoscopic stent removal

All endoscopic procedures were performed by experienced endoscopists or trainees under their direct supervision. Endoscopic stent removal was performed using either a duodenoscope or a single‐balloon enteroscope (JF‐260V, TJF‐260V, TJF‐Q290V, SIF‐H290S; Olympus Medical Systems, Tokyo, Japan) under conscious sedation with pethidine and midazolam. Before attempting stent removal or after attempting stent removal and experiencing resistance, biliary cannulation from the stent end or cannulation through the stent mesh was performed if possible. Balloon sweeping inside the stent was performed with stone extraction balloons under fluoroscopic guidance to check for contrast defects inside the stent suggesting tumor ingrowth. Rat‐tooth forceps, biopsy forceps, or a snare were used for stent removal at the discretion of the endoscopist. An attempt at stent removal was generally performed by grasping the stent with forceps or a snare, pushing the endoscope and twisting clockwise to move the stent slightly out of the bile duct, and regrasping further up on the stent near the ampulla to repeat the process. If unsuccessful, if strong resistance was felt, or if distal duodenal stenosis preclude pushing the endoscope, the stent was grasped and pulled together with the endoscope into the stomach. In either case, both the stent and the endoscope were carefully removed together from the patient's body under endoscopic and fluoroscopic guidance to avoid injury to the surrounding intestinal tract.

### Evaluation of clinical outcomes

Successful stent removal was defined as the complete removal of the stent from the bile duct. DMS was considered removed by pushing if any attempt was made to remove the stent by grasping it and pushing the endoscope and twisting clockwise, even if the stent was ultimately removed by pulling it into the stomach. DMS was considered removed by pulling into the stomach if this was the only method attempted, due to reasons such as duodenal stenosis. Procedure time was defined as the time from reaching the stent until successful stent removal or until the removal attempt was abandoned. Adverse events were graded according to the American Society of Gastrointestinal Endoscopy lexicon guidelines.^17^ If computed tomography showed only fluid densities inside the stent, it was defined as fluid‐filled. If it contained gas, it was defined as pneumobilia. Tumor ingrowth was defined as an obvious defect inside the stent on cholangiography after balloon sweeping (Figure S1). Inward and incomplete outward migrations were determined based on positional shifts in the upper edge of the stent confirmed on computed tomography, positional shifts of the distal stent tip confirmed on endoscopy, or both. Patients receiving biliary SEMS for the first time were defined as naïve cases, regardless of whether plastic stents or nasobiliary tubes had been placed in the past.

### In vitro study

Details on the experimental system of the in vitro study are summarized in Video S1. The experiment was conducted with two physicians (Yuto Yamada and Takashi Sasaki) and three staff members of SB‐Kawasumi Laboratories, Inc.

### Statistical analysis

Continuous variables are presented as medians (ranges) and were compared using the Mann‐Whitney U test. Categorical variables are described as absolute numbers (proportions) and were analyzed using the Chi‐squared or Fisher's exact test. Logistic regression analysis was performed to analyze factors affecting stent removal. The Mann‐Whitney U test and the Kruskal‐Wallis test were used to compare measurements in the in vitro study. A *p*‐value < 0.05 was considered statistically significant. All statistical analyses were performed with EZR ver. 1.53.^18^

## RESULTS

### Clinical evaluation

#### Patient characteristics

**FIGURE 2:**
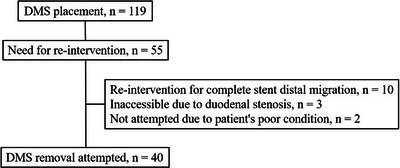
Patient flow diagram. DMS, duckbill‐type metal stent.

Forty patients were included in this study (Figure 2). Patient characteristics are summarized in Table 1. Most patients had metastatic pancreatic cancer (95%). Thirteen patients (32%) received DMS as a first stent and the remaining 27 patients (68%) had a history of previous CMS placement before DMS placement. The median indwelling period of DMS was 125 days (range 1–493). Stents were removed due to RBO in 36 patients (90%). Incomplete outward migration was observed in nine patients (23%) and inward migration was observed in seven patients (18%). Stent removal was necessary in all cases of incomplete outward migration (e.g., due to increased bile duct pressure due to sludge formation) or inward migration, despite not leading directly to RBO in many cases.

**TABLE 1:** Patient characteristics (*n* = 40).

Age, years	59 (41–85)
Sex, male/female	22 (55%)/18 (45%)
Performance status, 0/1/2	19 (48%)/17 (42%)/4 (10%)
Primary disease	
Pancreatic adenocarcinoma	38 (95%)
Pancreatic neuroendocrine carcinoma	1 (3%)
Intrahepatic cholangiocarcinoma (metastatic lymph node)	1 (3%)
Duodenal stenosis†, Type I/II/III	1 (3%)/6 (15%)/8 (20%)
Co‐existing duodenal metal stent	3 (8%)
Anti‐cancer treatment before stent removal	38 (95%)
Previous CMS before DMS placement	27 (68%)
Previous sphincterotomy	40 (100%)
Duodenoscope/single‐balloon enteroscope‡	39 (97%)/1 (3%)
Indwelling period of DMS, days	125 (1–493)
DMS length, 6 cm/8 cm	22 (55%)/18 (45%)
Fluid inside DMS on CT imaging	18 (45%)
Pneumobilia inside DMS on CT imaging	17 (42%)
Incomplete outward migration	9 (23%)
Inward migration§	7 (18%)
Reason for stent removal	
RBO	36 (90%)
Sludge/stone formation	18 (45%)
Food impaction	2 (5%)
Tumor ingrowth	5 (12%)
Non‐occlusive cholangitis	4 (10%)
Inward migration	5 (12%)
Incomplete outward migration	1 (3%)
Hemobilia	1 (3%)
Non‐RBO	4 (10%)
Conversion of biliary drainage route due to duodenal stenosis	2 (5%)
Obstructive pancreatitis	2 (5%)

Continuous variables are expressed as median (range) and categorical variables are expressed as absolute numbers (proportions).

Abbreviations: CMS, covered metal stent; CT, computed tomography; DMS, duckbill‐type metal stent; RBO, recurrent biliary obstruction.

^†^ Type classification for combined malignant biliary and duodenal obstruction was categorized according to the Mutignani classification.^19^

^‡^ One case involved DMS placement with a single‐balloon enteroscope in a patient with Roux‐en‐Y anastomosis.

^§^ Complete and incomplete migration towards the liver in five and two cases, respectively.

### Clinical outcomes of stent removal

Table 2 shows the clinical outcomes of stent removal. Stent removal was successfully achieved in thirty‐one cases (78%). No adverse events due to stent removal were noted.

**TABLE 2:** Clinical outcomes of stent removal (*n* = 40).

Devices used for stent removal	
Rat‐tooth forceps	25 (62%)
Snare	14 (35%)
Biopsy forceps	1 (3%)
Balloon sweeping inside DMS before stent removal	14 (35%)
Tumor ingrowth suspected during ERCP before attempting stent removal	2 (5%)
Tumor ingrowth suspected during ERCP after attempting stent removal	5 (12%)
Successful stent removal	31 (78%)
Procedure time, minutes	10 (3–35)
Successful cases	8 (4–35)
Unsuccessful cases	12 (3–34)
Main method of stent removal†	
By pushing the endoscope	22 (55%)
By pulling the endoscope into the stomach	18 (45%)
Stent tear during stent removal	14 (35%)
Additional procedures in unsuccessful cases	
CMS placement in DMS	7 (18%)
Plastic stent placement in DMS	1 (3%)
Stent trimming and balloon sweeping	1 (3%)
Adverse events due to stent removal	0 (0%)

Continuous variables are expressed as median (range) and categorical variables are expressed as absolute numbers (proportions).

Abbreviations: CMS, covered metal stent; DMS, duckbill‐type metal stent; RBO, recurrent biliary obstruction.

^†^ Stent removal in one case conducted using a single‐balloon enteroscope was considered to be performed “by pushing the endoscope” because force was applied parallel to the bile duct axis.

**FIGURE 3:**
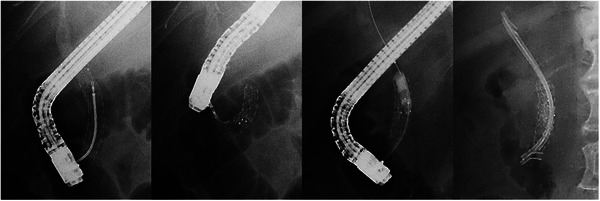
Failed stent removal using the invagination method.

Table 3 provides details of unsuccessful cases. Stent removal was unsuccessful in all seven cases with tumor ingrowth. One case had plastic stents placed inside the DMS after it was torn during the removal attempt, leaving the proximal portion of the DMS in the bile duct. The invagination method (grasping the top of the remaining portion with biopsy forceps) was attempted but led to the stent becoming stuck in an inverted position. We, therefore, used a balloon dilator to dilate the inverted DMS and placed two plastic stents inside the DMS (Figure 3). Although the stent could not be removed completely, biliary drainage was achieved without adverse events.

**TABLE 3:** Cases in which stent removal was unsuccessful.

Case	Primary disease	Duodenal stenosis	Naïve case	Indwelling period of DMS, days	Stent length, cm	CT findings	Migration	Reason for stent removal	Device	Tumor ingrowth	Method of stent removal	Stent tearing	Additional procedure
1	ICC (LN)	No	Yes	91	8	Pneumobilia	No	Slidge	Rat‐tooth forceps	NA	By pulling	Yes	PSs in DMS
2	PC	No	No	259	8	Fluid‐filled	Outward	Sludge	Snare	No	By pushing	Yes	Stent trimming and balloon sweeping
3	PC	No	Yes	469	8	Fluid‐filled	No	Sludge	Snare	Yes	By pulling	Yes	CMS in DMS
4	PC	No	Yes	270	8	Fluid‐filled	Outward	Tumor ingrowth	Snare	Yes	By pushing	No	CMS in DMS
5	PC	No	Yes	256	8	Pneumobilia	No	Sludge	Snare	Yes	By pulling	No	CMS in DMS
6	PC	Type II	Yes	124	8	Pneumobilia	No	Tumor ingrowth	Snare	Yes	By pushing	Yes	CMS in DMS
7	PC	No	Yes	283	8	Pneumobilia	No	Tumor ingrowth	Rat‐tooth forceps	Yes	By pushing	Yes	CMS in DMS
8	PC	No	Yes	125	6	NA	No	Tumor ingrowth	Rat‐tooth forceps	Yes	By pushing	No	CMS in DMS
9	PC	No	Yes	202	8	Pneumobilia	No	Tumor ingrowth	Rat‐tooth forceps	Yes	By pulling	Yes	CMS in DMS

Abbreviations: CMS, covered metal stent; CT, computed tomography; DMS, duckbill‐type metal stent; ICC, intrahepatic cholangiocarcinoma; LN, lymph node; N, number; NA, not available; PC, pancreatic cancer; PS, plastic stent.

Table 4 shows the results of the univariate logistic regression analysis. Naïve cases (OR [odds ratio], 41.60 [95% confidence interval [CI] 4.22–410.00]), longer stent (OR 16.80 [95% CI 1.84–153.00]), long indwelling time (OR 6.86 [95% CI 1.35–34.70]), and stent tearing during the removal procedure (OR 5.75 [95% CI 1.16–28.60]) were significantly associated with unsuccessful stent removal. Balloon sweeping inside DMS before stent removal was performed in only a small number of cases (14 cases), precluding statistical analyses on the effects of tumor ingrowth.

**TABLE 4:** Factors associated with unsuccessful stent removal.

				Univariate	
Factors		*n*	Unsuccessful cases	OR (95% CI)	*p*‐Value
Naïve papilla	Yes	13	8	41.60 (4.22–410.00)	<0.01
	No	27	1	ref	
Indwelling period of DMS	≥6 months	13	6	6.86 (1.35–34.70)	0.02
	<6 months	27	3	ref	
Stent length	8 cm	18	8	16.80 (1.84–153.00)	0.01
	6 cm	22	1	ref	
CT findings†	Fluid‐filled	18	3	0.48 (0.10–2.43)	0.36
	Pneumobilia	17	5	ref	
Stent migration	Yes	16	2	0.35 (0.06–1.94)	0.23
	No	24	6	ref	
Incomplete outward migration	Yes	9	2	0.98 (0.17–5.83)	0.98
	No	31	6	ref	
Removal device	Snare	14	5	3.06 (0.66–14.1)	0.15
	Rat‐tooth or biopsy forceps	26	4	ref	
Tumor ingrowth‡	Yes	7	7	NA	NA
	No	6	1		
Method of stent removal	By pulling	18	4	0.97 (0.22–4.32)	0.84
	By pushing	22	5	ref	
Stent tearing during stent removal	Yes	14	6	5.75 (1.16–28.60)	0.03
	No	26	3	ref	

Abbreviations: CI, confidence interval; CT, computed tomography; DMS, duckbill‐type metal stent; OR, odds ratio; NA, not available; ref, reference.

^†^ CT was not performed immediately before stent removal in five cases.

^‡^ Only evaluated by balloon sweeping and fluoroscopy in 14 cases, of which the presence of stent ingrowth could not be determined in one case due to failed extraction using the invagination method.

### Outcome of in vitro study

**FIGURE 4:**
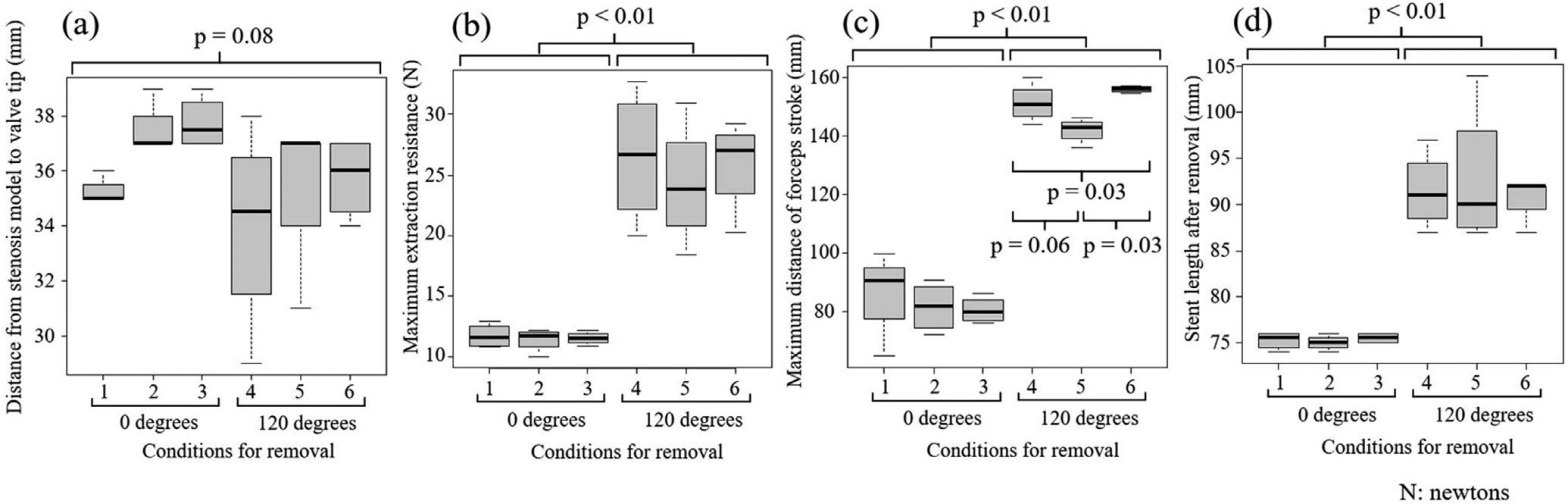
Comparison of results based on different conditions in the in vitro study, (a) Distance from stenosis model to valve tip. (b) Maximum extraction resistance. (c) Maximum distance of forceps stroke. (d) Stent length after removal.

Results are summarized in (Figure 4) and Table S1 Graphs of the relationship between extraction resistance and stroke distance required for extraction are provided in Figure S2 There was no significant difference in median length from stent tip to stenosis model between groups (*p =* 0.08). The median extraction resistance when applying force at 0° was lower (11.6 N [range 10.0–12.9] vs. 25.5 N [range 18.4–32.7], *p <* 0.01) and the median stroke distance was shorter (84.0 mm [range 64.7–99.5] vs. 150.7 mm [range 136.0–160.0], *p <* 0.01) than when applying force at 120°. The median stent length after removal was also shorter (75 mm [range 74–76] vs. 92 mm [range 87–104], *p <* 0.01), although one case in which the stent tore when grasped diagonally across multiple cells and removed with force applied at a 120° angle was excluded.

Among cases with force applied at 0°, no difference was observed between the three grasping conditions. Among those with force applied at 120°, there was a significant difference in the median maximum distance of forceps stroke between grasping methods (*p =* 0.03; Table S2). Specifically, the median distance of forceps stroke was shorter after being pulled with rat‐tooth forceps in a horizontal position than in a diagonal position (142.9 mm [range 136.0–146.3] vs. 156.0 mm [range 154.8–157.3], *p =* 0.03) or when pulled with a snare (150.7 mm [range 144.0–160.0], *p =* 0.06). Pulling diagonally with rat‐tooth forceps led to the most stent damage, with stent rupture in one case and stents in the other three cases being more severely damaged compared to the other conditions.

## DISCUSSION

In this study, DMS removal was successful in 78% (31/40) of cases. Tumor ingrowth was evident in most of the failed cases (78%, 7/9). Naïve cases, longer stents, long indwelling periods, and stent tearing during the removal procedure were also factors predicting unsuccessful stent removal.

Although there have been several reports on clinical outcomes of fully‐covered laser‐cut SEMS, ^5^, ^6^, ^7^, ^8^, ^20^, ^21^ few reports mention stent removal.^5^, ^6^, ^7^, ^8^ Marui et al.^5^ successfully removed fully‐covered laser‐cut SEMS with rat‐tooth forceps and a snare in eight of nine (89%) attempts. In the failed case, the stent was torn when pulled with rat‐tooth forceps. In a comparative study of fully‐covered laser‐cut and braided SEMS, successful stent removal rates between the two groups were comparable (88.9％ vs. 90％).^6^ The stent used in these studies has a double membrane structure of silicone and polyurethane, which has been suggested to be resistant to longitudinal traction and prevent tumor ingrowth.^6^ In a pilot feasibility study including nine cases, DMS removal was unsuccessful in three cases (33%) and the stent tore during the removal procedure in another three cases (33%).^7^

As the removal of laser‐cut DMSs can be difficult, it is important to understand situations in which stent removal can be challenging or impossible. In three previous reports investigating factors predicting successful SEMS removal, two reported that CMS was the only factor;^4^, ^22^ while the third reported that the length of the indwelling period may have an impact.^23^ We found that naïve cases, longer stents, long indwelling time, and stent tearing during the removal procedure were associated with difficult DMS removal. DMS removal in naïve cases may be more difficult than in cases where the stenosis has been pre‐dilated by previous CMSs. Longer stents are useful in preventing stent kinking, but there is a larger surface area of contact with the bile duct. Longer indwelling periods may be associated with tumor ingrowth. As stent tearing can also make stent removal difficult, techniques to avoid tearing are crucial. Forceful removal can only lead to tearing, but may also preclude further intervention due to entanglement. An alternative is to cut the ARV and place additional stents inside the DMS. We believe the invagination method should be avoided when removing laser‐cut SEMSs.

Although tumor ingrowth inside the stent was not evaluated in all patients, it plays a key role in predicting unsuccessful stent removal. As a method for judging tumor ingrowth, computed tomography and fluoroscopic findings were evaluated in addition to clinical information such as stent length and indwelling time of DMS. Balloon sweeping to evaluate defects inside DMS on fluoroscopy should be considered when possible before attempting removal.

In the in vitro study, experiments were conducted assuming several situations. As expected, the load applied to the stent was smaller when the extraction angle was 0° than when it was 120°. Thus, stress on the bile duct is smaller when DMSs are pushed parallel to the bile duct axis than when the endoscope is forcefully pulled into the stomach while grasping the stent. When the extraction angle was 0°, the choice of the endoscopic device had no significant impact on the force applied. However, when the extraction angle was 120°, grasping the stent horizontally with rat‐tooth forceps led to the lowest burden. Other gripping methods tended to tear the stent. DMS removal in cases with duodenal stenosis can be difficult,^24^ requiring the stent to be pulled into the stomach. If DMS needs to be removed under similar situations, it is better to grasp the stent wire horizontally with rat‐tooth forceps.

Based on the results of this study, our proposed strategy for DMS removal. The following factors were identified as predictors of removal difficulty: (1) tumor ingrowth, (2) naïve case, (3) longer stent, (4) longer indwelling time, and (5) stent tearing during removal. Because tumor ingrowth was the most important key factor, it is important to confirm the presence of tumor ingrowth at the time of stent removal. If the DMS is placed normally across the papilla, cannulation should be tried from the stent end and the tumor ingrowth should be evaluated by cholangiography with balloon sweeping. If the DMS is migrating outward, cannulation through the stent mesh is attempted to assess tumor ingrowth. In the case of inward migration, it is sometimes difficult to assess tumor ingrowth. The stent that has migrated into the bile duct must be withdrawn into the duodenum using forceps. Although tumor ingrowth is not usually observed in such cases, an additional stent should be considered instead of attempting removal with excessive force if there is resistance to stent removal. When no tumor ingrowth was suggested before stent removal in either case, the possibility of stent removal is evaluated based on clinical information such as the naïve case, stent length, and duration of stent placement. If these factors are present and there is resistance to stent removal, stent removal should be discontinued and an additional stent placement should be considered. When DMS has to be pulled into the stomach for stent removal, rat‐tooth forceps should be selected, and grasping stent wires horizontally may prevent stent tearing. Since there is a risk of bleeding due to forceful stent removal, it is necessary to try to perform the procedure as gently as possible.^25^ When stent removal is successful, a new SEMS can be placed for biliary drainage.

We acknowledge several limitations in this study. First, this was a retrospective study from a single institution. Although this was the largest report on stent removal of laser‐cut CMS, the sample size was not sufficient to permit multivariate analysis. Second, the strategy of stent removal had not been standardized. Tumor ingrowth was not assessed in a majority of cases and its effect on stent removal could not be analyzed. Third, our experimental system is an extremely simplified model, and care is warranted when applying the results of this experiment to real‐world practice.

In conclusion, DMS was safely removed in a majority of cases. Deployment of additional stents inside DMS may be preferable to forceful removal in the presence of factors associated with difficult stent removals, such as tumor ingrowth, naïve cases, longer stents, long indwelling time, and stent tearing during the removal procedure.

## CONFLICT OF INTEREST STATEMENT

Three staff members of SB‐Kawasumi Laboratories, Inc (Keisuke Yatsuo, Tomoaki Yokota, and Tomokazu Mukai) conducted in vitro study with two doctors (Yuto Yamada and Takashi Sasaki). All these staff members of SB‐Kawasumi Laboratories, Inc were not involved in data interpretation or manuscript writing. The authors except for the staff members of SB‐Kawasumi Laboratories, Inc declare no conflict of interest.

## Supporting information


**Supplementary Figure 1**. (a) Fluid‐filled inside DMS. (b) Pneumobilia inside DMS. (c) Tumor ingrowth suspected on fluoroscopy after balloon sweeping (arrow).

**Supplementary Figure 2**. Graphs showing the relationship between extraction resistance value and the stroke distance of forceps required for extraction. X‐axis: extraction resistance value (N); Y‐axis: forceps stroke distance (mm). Clear differences were observed with different extraction angles, but not with choice of extraction device or grasping method.

**Supplementary Table 1**. Outcomes of the in vitro study.

**Supplementary Table 2**. Comparison of results based on extraction angle.

**Supplementary Video 1**. Experimental system of the in vitro study.

## References

[1] Almadi MA , Barkun A , Martel M . Plastic vs. self‐expandable metal stents for palliation in malignant biliary obstruction: A series of meta‐analyses. Am J Gastroenterol 2017;112:260–73. 27845340 10.1038/ajg.2016.512

[2] Tringali A , Hassan C , Rota M Rossi, M , Mutignani M , Aabakken L . Covered vs. uncovered self‐expandable metal stents for malignant distal biliary strictures: A systematic review and meta‐analysis. Endoscopy 2018;50:631–41. 29342491 10.1055/s-0043-125062

[3] Yamashita Y , Tachikawa A , Shimokawa T *et al*. Covered versus uncovered metal stent for endoscopic drainage of a malignant distal biliary obstruction: Meta‐analysis. Dig Endosc 2022;34:938–51. 35114036 10.1111/den.14260

[4] Familiari P , Bulajic M , Mutignani M *et al*. Endoscopic removal of malfunctioning biliary self‐expandable metallic stents. Gastrointest Endosc 2005;62:903–10. 16301035 10.1016/j.gie.2005.08.051

[5] Marui S , Uza N , Yamazaki H *et al*. Utility of laser‐cut covered self‐expandable metal stents for unresectable malignant distal biliary obstruction: A single‐center experience. Endoscopy 2020;52:664–8. 32316040 10.1055/a-1149-1700

[6] Tanisaka Y , Mizuide M , Fujita A *et al*. Can the laser‐cut covered self‐expandable metallic stent be the first choice for patients with unresectable distal malignant biliary obstruction? (with video). J Hepatobiliary Pancreat Sci 2022;29:585–93. 34390208 10.1002/jhbp.1034

[7] Kin T , Ishii K , Okabe Y , Itoi T , Katanuma A . Feasibility of biliary stenting to distal malignant biliary obstruction using a novel designed metal stent with duckbill‐shaped anti‐reflux valve. Dig Endosc 2021;33:648–55. 32875614 10.1111/den.13827

[8] Yamada Y , Sasaki T , Takeda T *et al*. A novel laser‐cut fully covered metal stent with anti‐reflux valve in patients with malignant distal biliary obstruction refractory to conventional covered metal stent. J Hepatobiliary Pancreat Sci 2021;28:563–71. 33835728 10.1002/jhbp.966

[9] Sasaki T , Takeda T , Sasahira N . Double stenting with EUS‐CDS using a new anti‐reflux metal stent for combined malignant biliary and duodenal obstruction. J Hepatobiliary Pancreat Sci 2020;27:e15–6. 32846046 10.1002/jhbp.818

[10] Sasaki T , Takeda T , Yamada Y *et al*. Long‐term outcomes of endoscopic double stenting using an anti‐reflux metal stent for combined malignant biliary and duodenal obstruction. J Hepatobiliary Pancreat Sci 2023;30:144–52. 35583159 10.1002/jhbp.1181

[11] Hinokuchi M , Hashimoto S , Kojima I *et al*. Efficacy and safety of a novel anti‐reflux metal stent during neoadjuvant chemotherapy for pancreatic cancer: A prospective multicenter exploratory study. J Hepatobiliary Pancreat Sci. Published online: 15 Sep 2022; DOI: 10.1002/jhbp.1239 36106919

[12] Koga T , Hijioka S , Ishikawa Y *et al*. Duckbill‐type antireflux self‐expandable metal stent placement for post‐choledochojejunostomy reflux cholangitis. Endoscopy 2021;53:E174–6. 32818991 10.1055/a-1216-1220

[13] Renno A . Antireflux valve metal stent versus conventional self‐expandable metal stent in distal malignant biliary obstruction: A systematic review and meta‐analysis. Ann Gastroenterol 2019;32:605–13. 31700238 10.20524/aog.2019.0427 PMC6826073

[14] Isayama H , Hamada T , Yasuda I *et al*. TOKYO criteria 2014 for transpapillary biliary stenting. Dig Endosc 2015;27:259–64. 25209944 10.1111/den.12379

[15] Hamada T , Nakai Y , Isayama H . TOKYO criteria: Standardized reporting system for endoscopic biliary stent placement. Gastrointest Interv 2018;7:46–51.

[16] Togawa O , Isayama H , Tsujino T *et al*. Management of dysfunctional covered self‐expandable metallic stents in patients with malignant distal biliary obstruction. J Gastroenterol 2013;48:1300–7. 23354625 10.1007/s00535-013-0751-z

[17] Cotton PB , Eisen GM , Aabakken L *et al*. A lexicon for endoscopic adverse events: Report of an ASGE workshop. Gastrointest Endosc 2010;71:446–54. 20189503 10.1016/j.gie.2009.10.027

[18] Kanda Y . Investigation of the freely available easy‐to‐use software ‘EZR’ for medical statistics. Bone Marrow Transplant 2013;48:452–8. 23208313 10.1038/bmt.2012.244 PMC3590441

[19] Mutignani M , Tringali A , Shah SG *et al*. Combined endoscopic stent insertion in malignant biliary and duodenal obstruction. Endoscopy 2007;39:440–7. 17516351 10.1055/s-2007-966327

[20] Isayama H , Kawakubo K , Nakai Y *et al.* A novel, fully covered laser‐cut nitinol stent with antimigration properties for nonresectable distal malignant biliary obstruction: A multicenter feasibility study. Gut Liver 2013;7:725–30. 24312715 10.5009/gnl.2013.7.6.725 PMC3848551

[21] Kitagawa K , Mitoro A , Ozutsumi T *et al.* Laser‐cut‐type versus braided‐type covered self‐expandable metallic stents for distal biliary obstruction caused by pancreatic carcinoma: A retrospective comparative cohort study. Clin Endosc 2022;55:434–42. 34706489 10.5946/ce.2021.161 PMC9178141

[22] Shin HP , Kim MH , Jung SW *et al*. Endoscopic removal of biliary self‐expandable metallic stents: A prospective study. Endoscopy 2006;38:1250–5. 17163328 10.1055/s-2006-944969

[23] Ishii K . Endoscopic removal and trimming of distal self‐expandable metallic biliary stents. World J Gastroenterol 2011;17:2652–7. 21677835 10.3748/wjg.v17.i21.2652 PMC3110929

[24] Sasaki T , Takeda T , Sasahira N . Removal of a biliary self‐expandable metal stent using the zipline technique for pancreatic cancer with duodenal stricture. Dig Endosc 2022;34:e26–7. 34845764 10.1111/den.14190

[25] Tanisaka Y , Mizuide M , Fujita A *et al*. Hemorrhage after laser‐cut covered self‐expandable metal stent removal. Endoscopy 2022;54:E378–9. 34374056 10.1055/a-1541-7205

