# Supplementary figures and images for: Removal of Duckbill‐type laser‐cut anti‐reflux metal stents: Clinical evaluation and in vitro study
Source: DEN Open. 2023 Feb 23;3(1):e217. doi: 10.1002/deo2.217 (PMC9950539; doi:10.1002/deo2.217)

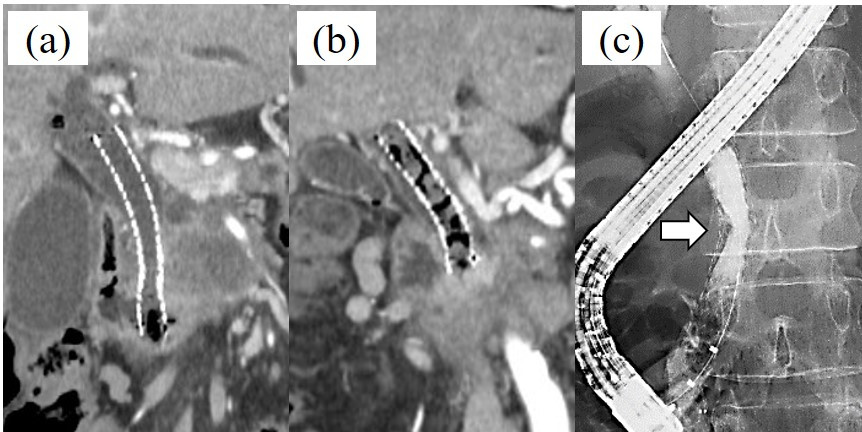

Supplement: Supplementary file 1 — Supplementary Figure 1. (a) Fluid‐filled inside DMS. (b) Pneumobilia inside DMS. (c) Tumor ingrowth suspected on fluoroscopy after balloon sweeping (arrow). [file DEO2-3-e217-s004.jpg]

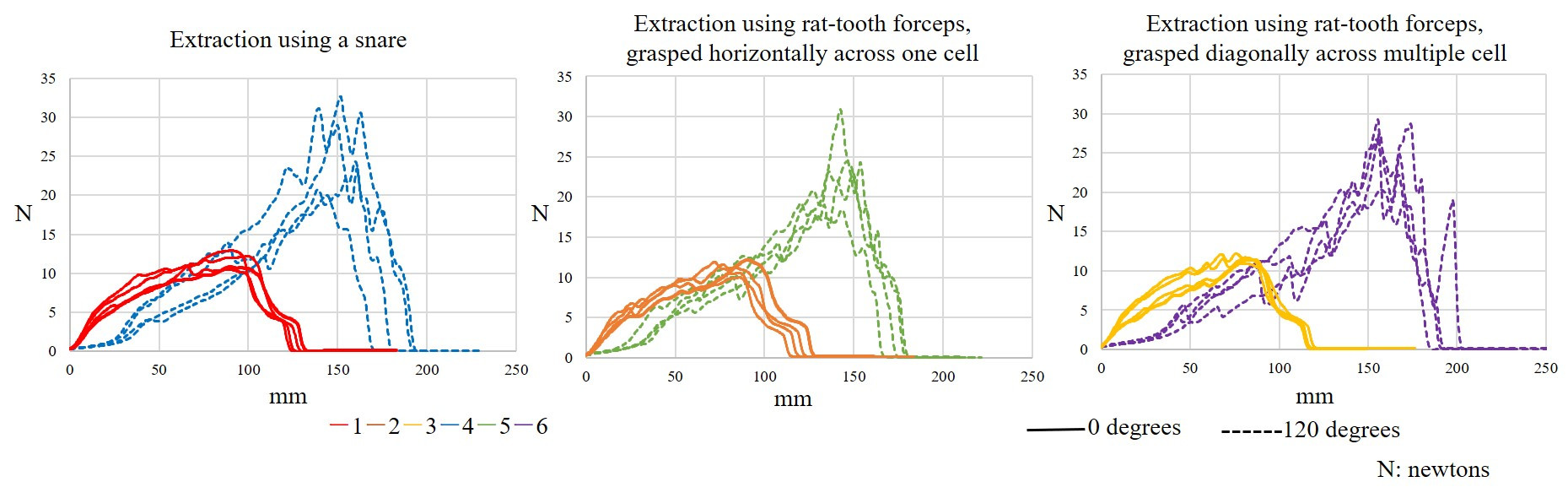

Supplement: Supplementary file 2 — Supplementary Figure 2. Graphs showing the relationship between extraction resistance value and the stroke distance of forceps required for extraction. X‐axis: extraction resistance value (N); Y‐axis: forceps stroke distance (mm). Clear differences were observed with different extraction angles, but not with choice of extraction device or grasping method. [file DEO2-3-e217-s002.jpg]
