# Supplementary material for: Removal of Duckbill‐type laser‐cut anti‐reflux metal stents: Clinical evaluation and in vitro study
Source: DEN Open. 2023 Feb 23;3(1):e217. doi: 10.1002/deo2.217 (PMC9950539; doi:10.1002/deo2.217)
Supplement: Supplementary file 3 — Supplementary Table 1. Outcomes of the in vitro study. [file DEO2-3-e217-s001.docx]

**Supplementary table 1.** Outcomes of the *in vitro* study

| Conditions | Extraction angle | Device | Grasping method | Distance from stenosis model to valve tip, mm | Maximum extraction resistance, N | Maximum distance of forceps stroke, mm | Stent length after removal, mm |
| --- | --- | --- | --- | --- | --- | --- | --- |
| 1 | 0° | Snare | Between cells | 35 | 10.8 | 90.0 | 75 |
|  |  |  |  | 36 | 12.2 | 99.5 | 76 |
|  |  |  |  | 35 | 12.9 | 90.7 | 76 |
|  |  |  |  | 35 | 11.0 | 64.7 | 74 |
| 2 |  | Rat-tooth forceps | Horizontally across  one cell | 37 | 12.2 | 90.7 | 75 |
|  |  |  |  | 37 | 11.9 | 72.0 | 76 |
|  |  |  |  | 39 | 10.0 | 86.2 | 74 |
|  |  |  |  | 37 | 11.6 | 76.7 | 75 |
| 3 |  |  | Diagonally across multiple cells | 37 | 11.4 | 86.3 | 76 |
|  |  |  |  | 39 | 11.6 | 81.8 | 76 |
|  |  |  |  | 37 | 12.2 | 76.0 | 75 |
|  |  |  |  | 38 | 10.9 | 77.6 | 75 |
| 4 | 120° | Snare | Between cells | 29 | 24.3 | 160.0 | 90 |
|  |  |  |  | 34 | 32.7 | 151.5 | 97 |
|  |  |  |  | 38 | 29.0 | 149.8 | 92 |
|  |  |  |  | 35 | 20.0 | 144.0 | 87 |
| 5 |  | Rat-tooth forceps | Horizontally across  one cell | 37 | 24.4 | 146.3 | 88 |
|  |  |  |  | 31 | 23.2 | 136.0 | 92 |
|  |  |  |  | 37 | 30.9 | 142.5 | 104 |
|  |  |  |  | 37 | 18.4 | 143.3 | 87 |
| 6 |  |  | Diagonally across multiple cells | 34 | 27.4 | 156.5 | 92 |
|  |  |  |  | 37 | 29.2 | 154.8 | 92 |
|  |  |  |  | 35 | 26.6 | 157.3 | 87 |
|  |  |  |  | 37 | 20.3 | 155.5 | Torn |

N, newtons
