# Supplementary material for: Removal of Duckbill‐type laser‐cut anti‐reflux metal stents: Clinical evaluation and in vitro study
Source: DEN Open. 2023 Feb 23;3(1):e217. doi: 10.1002/deo2.217 (PMC9950539; doi:10.1002/deo2.217)
Supplement: Supplementary file 4 — Supplementary Table 2. Comparison of results based on extraction angle. [file DEO2-3-e217-s005.docx]

**Supplementary table 2.** Comparison of results based on extraction angle

| Conditions | 1 | 2 | 3 | *p value* |
| --- | --- | --- | --- | --- |
| Extraction angle | 0° | | |  |
| Device | Snare | Rat-tooth forceps | |  |
| Grasping method | Between cells | Horizontally across one cell | Diagonally across multiple cells |  |
| Distance from stenosis model to valve tip, mm, median (range) | 37 (35–38) | 37 (37–39) | 37.5 (37–39) | *0.51* |
| Maximum extraction resistance, N, median (range) | 11.6 (10.8–12.9) | 11.8 (10.0–12.2) | 11.5 (10.9–12.2) | *0.94* |
| Maximum distance of forceps stroke, mm, median (range) | 90.4 (64.7–99.5) | 81.5 (72.0–90.7) | 79.7 (76.0–86.3) | *0.54* |
| Stent length after removal, mm, median (range) | 76 (74–76) | 75 (74–76) | 76 (75–76) | *0.66* |

| Conditions | 4 | 5 | 6 | *p value* |
| --- | --- | --- | --- | --- |
| Extraction angle | 120° | | |  |
| Device | Snare | Rat-tooth forceps | |  |
| Grasping method | Between cells | Horizontally across one cell | Diagonally across multiple cells |  |
| Distance from stenosis model to valve tip, mm, median (range) | 35 (29–38) | 37 (31–37) | 36 (34–37) | *0.76* |
| Maximum extraction resistance, N, median (range) | 26.7 (20–32.7) | 23.8 (18.4–30.9) | 27.0 (20.3–29.2) | *0.79* |
| Maximum distance of forceps stroke, mm, median (range) | 150.7 (144.0–160.0) | 142.9 (136.0–146.3) | 156.0 (154.8–157.3) | *0.03* |
| Stent length after removal, mm, median (range) | 91 (87–97) | 90 (87–104) | 92 (87–92)† | *0.98* |

†One case of stent tearing was excluded.
